# Epidemiology of Bedbound Status at the End of Life in a National Sample of Older Adults

**DOI:** 10.1093/geroni/igaf122.1029

**Published:** 2025-12-31

**Authors:** Katherine Ornstein, Mary Lou Pomeroy, Hanna Charankevich, Po-Jen Kung, Bruce Leff, Jennifer Reckrey

**Affiliations:** Johns Hopkins University, Baltimore, Maryland, United States; Johns Hopkins University, Baltimore, Maryland, United States; Johns Hopkins University, Baltimore, Maryland, United States; Johns Hopkins University, Baltimore, Maryland, United States; Johns Hopkins University School of Medicine, Baltimore, Maryland, United States; NYU Grossman School of Medicine, New York, New York, United States

## Abstract

As the number of individuals living in the community at the end of life grows, many will become bedbound. This may be especially true for individuals with dementia, who have extensive care needs. Using the National Health and Aging Trends Study (NHATS), we examined bedbound status up to 12 months before death among 3,459 community-dwelling individuals ages 65+ who died between 2012–2023. We defined bedbound status using self/proxy reports of frequency, assistance, and difficulty leaving the bed/bedroom. Probable dementia status was defined using an NHATS algorithm that includes cognitive testing and diagnosis history. We estimated the predicted probability of being bedbound by dementia status using survey weights and adjusting for sociodemographic, clinical, and health characteristics. We also examined weekly hours of help received by the bedbound in the 12 months before death. Nearly 2.5 million decedents were bedbound in their last year of life. Individuals with dementia had an increased likelihood of being bedbound (Predicted probability = 23%) and required extensive help (Mean = 3.5 helpers averaging 123 hours/week in the month before death). This is the first national study of bedbound status at the end of life among older adults. A considerable portion of individuals with dementia are confined to bed in the last year of life, requiring extensive help with self-care and other activities. The high number of bedbound individuals with dementia at the end of life has important implications for the need for paid and unpaid caregiving support, hospice, and other home-based care services.

